# Complete Genome Sequence of Porcine Deltacoronavirus Strain CH/JXJGS01/2016, Isolated in Jiangxi Province, China, 2016

**DOI:** 10.1128/genomeA.00832-17

**Published:** 2017-08-24

**Authors:** Min Zhang, Yu Ye, Wang Gong, Nannan Guo, Fanfan Zhang, Anqi Li, Xingrong Zhou, Dongyan Huang, Deping Song, Yuxin Tang

**Affiliations:** Key Laboratory for Animal Health of Jiangxi Province, Nanchang, Jiangxi, China, and Department of Preventive Veterinary Medicine, College of Animal Science and Technology, Jiangxi Agricultural University, Nanchang, Jiangxi, China

## Abstract

The complete genome sequence of a variant of porcine deltacoronavirus, isolated from a diarrheal piglet and designated CH/JXJGS01/2016, was sequenced and analyzed. Phylogenetic analysis demonstrated that CH/JXJGS01/2016 shares the highest nucleotide and amino acid identities with the Chinese strain NH (GenBank accession number KU981059).

## GENOME ANNOUNCEMENT

Porcine deltacoronavirus (PDCoV) is an enveloped single-stranded positive-sense RNA virus that belongs to the newly classified genus *Deltacoronavirus*, in the family *Coronaviridae* (1–3). PDCoV was first reported in pig feces in Hong Kong in 2012, and in the United States, it was initially recognized in pigs suffering from severe diarrhea in early 2014 (4–6). In China, PDCoV has been frequently detected in diarrheal pigs, especially in newborn piglets with watery diarrhea caused by porcine epidemic diarrhea virus (PEDV) (7). In this study, the full-length genome sequence of a variant of PDCoV was identified and sequenced from a neonatal piglet suffering from a sudden outbreak of watery diarrhea in Jiangxi, China, in 2016.

A variant of PDCoV, designated CH/JXJGS01/2016, was isolated from one of the PDCoV-positive samples by our laboratory. The viral RNA was extracted from the suspensions of virus cultured on a pig kidney cell line (LLC-PK1), and then the whole-genome sequence of CH/JXJGS01/2016 was amplified and sequenced based on the methods that we previously reported (8). Sequences were assembled into the full-length genome of CH/JXJGS01/2016 using Lasergene version 7.0 software (DNAStar, Inc., Madison, WI, USA). Phylogenetic analysis of the genome was performed with MEGA version 6.02 software (http://www.megasoftware.net) (9).

The complete genome of CH/JXJGS01/2016 is 25,438 nucleotides (nt) in length, excluding the 3′ poly(A) tail. The genome organization, which resembles that of all previously reported PDCoV genomes in GenBank, was ordered as follows: 5′ untranslated region (UTR), open reading frame 1a/b (ORF1a/b), spike (S), envelope (E), membrane (M), nonstructural protein 6 (NS6), nucleocapsid (N), nonstructural protein 7 (NS7), and 3′ UTR. The nucleotide locations were as follows: ORF1a/b, nt 540 to 19342; S gene, nt 19324 to 22803; E gene, nt 22787 to 23048; M gene, nt 23041 to 23694; N gene, nt 23999 to 25074; NS6 gene, nt 23694 to 23978; and NS7 gene, nt 24093 to 24695.

A homology analysis was carried out based on the full-length genome sequence and S gene of CH/JXJGS01/2016 and the reference strains of PDCoV retrieved from GenBank. The results revealed that CH/JXJGS01/2016 had the closest relationship to and highest nucleotide identity (99.4%) with a Chinese PDCoV strain, NH (GenBank accession number KU981059), and had the lowest nucleotide identity with two strains from Thailand (KU984334 and KU051641, 96.7%). The phylogenetic tree of the S gene indicated that CH/JXJGS01/2016 and strain NH were clustered in the same clade. Furthermore, CH/JXJGS01/2016 had two amino acid (aa) deletions at aa positions 51 and 52, compared with the S genes of the PDCoVs from the United States.

The full-length genome sequence of CH/JXJGS01/2016 determined in this study will increase our knowledge of PDCoV virology, providing a basis for the development of diagnostic tools and vaccine candidates. Further in-depth phylogenetic analysis of PDCoV requires more complete genome sequences of PDCoVs originating worldwide.

### Accession number(s).

The complete genome sequence of PDCoV strain CH/JXJGS01/2016 has been deposited in GenBank under the accession number KY293677.
