# Correction for Zhang et al., “Complete Genome Sequence of Porcine Deltacoronavirus Strain CH/JXJGS01/2016, Isolated in Jiangxi Province, China, 2016”

**DOI:** 10.1128/genomeA.01075-17

**Published:** 2017-09-28

**Authors:** Min Zhang, Qi Peng, Yu Ye, Wang Gong, Nannan Guo, Fanfan Zhang, Anqi Li, Xingrong Zhou, Dongyan Huang, Deping Song, Yuxin Tang

**Affiliations:** Key Laboratory for Animal Health of Jiangxi Province, Nanchang, Jiangxi, China, and Department of Preventive Veterinary Medicine, College of Animal Science and Technology, Jiangxi Agricultural University, Nanchang, Jiangxi, China

## AUTHOR CORRECTION

Volume 5, no. 34, e00832-17, 2017, https://doi.org/10.1128/genomeA.00832-17. Page 1: The article byline should read as given above.

Page 1: The equal-contribution footnote should read as follows. “M.Z., Q.P., and Y.Y. contributed equally to this work.”

